# Expression of MSTN/Smad signaling pathway genes and its association with meat quality in Tibetan sheep (*Ovis aries*)

**DOI:** 10.1002/fsn3.3216

**Published:** 2023-01-18

**Authors:** Na He, Xia Lang, Cailian Wang, Cailing Lv, Mingming Li, Ruizhe Sun, Junxia Zhang

**Affiliations:** ^1^ College of Agriculture and Animal Husbandry/Key Laboratory of Livestock and Poultry Genetics and Breeding on the Qinghai‐Tibet Plateau, Ministry of Agriculture and Rural Affairs/Plateau Livestock Genetic Resources Protection and Innovative Utilization Key Laboratory of Qinghai Province Qinghai University Xining Qinghai China; ^2^ Gansu Key Laboratory of Cattle and Sheep Germplasm and Straw Fodder Gansu Academy of Agricultural Sciences Lanzhou China

**Keywords:** gene expression, MSTN/Smad signaling pathway, mutton quality, Tibetan sheep

## Abstract

Tibetan sheep is a unique breed living in Qinghai‐Tibet Plateau. Since MSTN/Smad signaling pathway plays a critical role in the regulation of muscle development, we aimed to study the mutton quality, mRNA expression of main transduction genes in the MSTN/Smad signaling pathway, and the effects of those genes on the mutton quality of Tibetan sheep in this study. Six‐month‐old Qinghai‐Tibetan sheep were selected, slaughtered, and their Longissimus lumborum, semitendinosus muscle, arm triceps, and quadriceps femoris muscle were collected. The mutton quality was evaluated, and gene expression and their association with the mutton quality were analyzed using RT‐qPCR. The results showed that the indexes of mutton quality were not significantly different between ewes and rams (*p >* .05) except for Warner–Bratzler shear force (WBSF) (*p* < .05). A total of 21 different fatty acids were detected in the muscles of Tibetan sheep, including nine types of SFA, four types of MUFA, and eight types of PUFA. The main transduction genes of the MSTN/Smad signaling pathway were found to be widely expressed in muscle tissues, but no significant differences were observed (*p >* .05). The correlation analysis of the main genes and mutton quality showed that *MSTN* was significantly correlated with redness and cooking time; *Smad*2, *Smad*3, *Smad*4, and *TGFβRI* had significant positive correlations with marbling in arm triceps; *Smad*3 and *TGFβRII* had strong negative correlations with pH_24 h_ in Longissimus lumborum; *Smad*2 was negatively correlated with drip loss in Longissimus lumborum. In short, the expression level of *MSTN* in muscles was positively correlated with *Smad*2, *Smad*3, and *Smad*4 genes and negatively correlated with *TGFβRII* genes. Thus, the results of this study provide a theoretical basis for the regulation mechanism of the MSTN/Smad pathway on mutton quality.

## Graphical abstract

In this paper, the expression profiles of major transduction genes (*MSTN*, *Smad2*, *Smad3*, *Smad4*, *TGFBR1*, and *TGFBR2*) in the MSTN/Smad signaling pathway in Tibetan sheep muscle tissues are detected by real‐time fluorescence quantitative PCR, to understand the expression patterns and characteristics of major transduction genes of MSTN/Smad signaling pathway in Tibetan sheep muscle tissue and through the evaluation of Tibetan mutton quality, our results may provide theoretical evidence for the research on the regulation mechanism of MSTN/Smad signal pathway on mutton quality, but also push forward the improvement of molecular breeding level in Tibetan sheep.

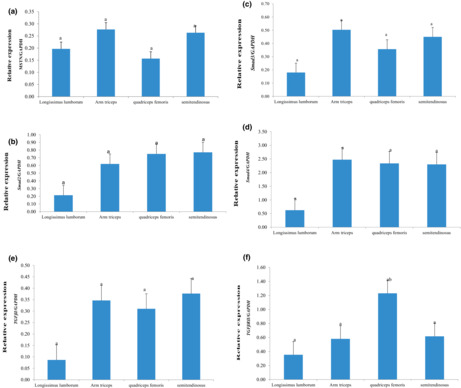

## 1 INTRODUCTION

Tibet has a history of more than 4000 years and Tibetan sheep are an excellent Chinese indigenous breed mainly distributed in the unique ecological environment of the Qinghai‐Tibet Plateau, the world's highest and largest plateau (Lv et al., 2018). The central breeding region is located in the southwest region of the plateau. Since Tibetan sheep live in highlands with an average altitude of >4000 m, they are well adapted to the harsh conditions, such as low oxygen, low temperatures, strong ultraviolet radiation, and severe seasonal imbalances in forage supply (Lu et al., 2021; Lv et al., 2018). This breed has unique and distinctive characteristics at the morphological, physiological, and genetic levels. Tibetan sheep graze on natural pasture all year round without supplementary feed (Jing et al., 2022). Consequently, Tibetan mutton is more popular among local consumers because it is rich in nutrition, low in cholesterol, delicious in taste, and has less odor. The Tibetan sheep industry is recently showing an extensive market prospect (Frick et al., 2017).

Genes of the MSTN/Smad signaling pathway play a crucial role in mammalian embryo development and the regeneration process of skeletal muscle (Wang et al., 2008). *Smad* signal transduction consists of extracellular ligands, specific receptors on the cell surface, and Smad molecules. Myostatin binds to the activin type II receptor (*ActRIIB*) and induces its assembly with the activin type I receptor. Subsequently, *Smad*2 and *Smad*3, upon phosphorylation, bind to *Smad*4, and the whole complex is translocated to the nucleus for repressing the transcription of genes associated with muscle formation (Elkina et al., 2011). Consequently, a tight connection is established among the three reactions, which ultimately delivers extracellular signals to the nucleus for controlling the transcription of the target gene, thereby activating the associated biological effects (Thomas et al., 2000). Signaling in this pathway involves a large number of molecules like TGF‐β, BMP, and hormones. Many *Smad* proteins like five specific glandular receptors (R‐*SMAD*S: *Smad*1, *Smad*2, *Smad*3, *Smad*5, and *Smad*8), one universal *Smad* (*Smad*4), and two regulatory *Smads* (*Smad*6 and *Smad*7) are also involved in this pathway. *Smad* signal transduction is a dynamic process that moves between the nucleus and plasma and is mainly regulated by ligands (Altomare, 2010).

Myostatin (*MSTN*) can regulate quantitative characteristics of muscles, is a negative regulator of muscle growth, and is used as an indicator of meat quality and livestock yield (Bi et al., 2020). Gay et al. found that myostatin is directly involved in the control of neuromuscular interrelationships or indirectly impacts muscle size (Gay et al., 2012). Furthermore, mutations in the *MSTN* gene are seen in many species, such as cattle and sheep, exhibiting the “double muscle” phenomenon (Aiello et al., 2018). In 1997, researchers at Hopkins University accidentally discovered the *MSTN* gene in mice and confirmed it as a member of the TGF‐β superfamily. Pragada et al. showed that *MSTN* is related to *Smad*3 in its function as a negative regulator of skeletal muscle growth. *MSTN* inhibits the expression and activation of myogenic regulatory factors through *Smad*3 and inhibits the differentiation of myoblasts into myotubes (Rebbapragada et al., 2003). Earlier studies have shown that *MSTN* is expressed in many tissues. Roberts & Goetz have reported the expression of *MSTN* in adult piscine skeletal muscle (Roberts & Goetz, 2001). Anwar et al. have found that the *MSTN* gene is expressed in triceps femoris, biceps femoris, and Longissimus lumborum of Xinjiang Turpan black lamb and Altay lamb. It is consistent with the finding that the *MSTN* gene negatively regulates muscle growth (Anwar et al., 2016; Hamutai et al., 2014).

Smad proteins are mediators that transmit signals from the cytoplasm to the nucleus and regulate the transcription of target genes. *Smad*2 and *Smad*3 are the direct substrates of receptor kinases that modulate endocrine signaling (Zhu, 2006). Rehman et al. have confirmed that the expression of the *Smad*2 gene is higher in skeletal muscle than in smooth muscle (Hamutai et al., 2014). Lin et al. conducted a correlation analysis on gene expression in different skeletal muscle tissues of Hu sheep and found that *Smad*3 gene expression is affected by different tissues, growth stages, and gender (Lin et al., 2015). Smad4 protein mediates the transcription of TGF‐β responsive genes. *Smad*4 protein participates in the signal transduction of all members of the TGF‐β superfamily and plays a crucial role in the growth of germ cells (Ahmed et al., 2017). Bao et al. have reported that *Smad*4 being the central molecule of this signaling pathway is essential in testicular reproductive organs and related reproductive tissues (Bao et al., 2016). *TGFβR I* and *TGFβRII* are members of the transforming growth factor superfamily **(**TGF‐β) and have been studied intensively. Earlier studies have focused primarily on the biological functions of TGF‐β family members related to medicine and embryo development. In recent years, the research has expanded to functions such as cell growth, cloning, and genetic breeding. The TGF‐β signaling pathway regulates a variety of cellular responses associated with animal growth and development, and the genes involved in this pathway have been widely studied in cattle, sheep, and pigs (Cai et al., 2019).

In summary, the *MSTN* gene can inhibit muscle growth and differentiation by regulating associated growth factors and proteins. Nevertheless, the molecular composition and regulation of the MSTN/Smad signal transduction pathway are complex due to the involvement of different regulatory factors. Consequently, the regulation mechanism and the gene expression pattern of this pathway need to be studied further. In this study, the expression profiles of major transduction genes (*MSTN*, *Smad*2, *Smad*3, *Smad*4, *TGFβRI*, and *TGFβRII*) of the MSTN/Smad signaling pathway in Tibetan sheep muscle tissues were studied by real‐time fluorescence quantitative PCR, and the correlation between their expression and meat quality were evaluated. This study may provide theoretical evidence for the regulation of mutton quality by the MSTN/Smad signal pathway, which may push forward the improvement of molecular breeding methods in Tibetan sheep.

## 2 MATERIALS AND METHODS

### 2.1 Animals and sampling

Tibetan sheep were sampled from an abattoir (Qinghai Xiangkameiduo Animal Husbandry Co. Ltd). Six‐month‐old Tibetan sheep of comparable weight and health were randomly selected, followed by their slaughter (three ewes, three rams). The meat was graded, and muscle tissues, including the longissimus dorsi, semitendinosus, arm triceps, and quadriceps femoris, were collected within 20 min of slaughter, and placed in the cryopreservation tube. These tubes were quickly placed into the liquid nitrogen tank and immediately frozen at −80°C until further analysis.

### 2.2 Measurement of meat quality

The fresh meat was cut into approximately 1‐cm‐thick slices. Meat slices were kept at room temperature, and the meat color was measured by Minolta CR 300, and color assessment was performed using CIELAB. The results were expressed as lightness (L*), redness (a*), and yellowness (b*), calibrated against a standard white tile (8‐mm‐diameter aperture, D65 illuminant, and 10° standard observer angle), after 30 min of slaughter at three positions on the surface of the cross‐section of Longissimus lumborum after exposing the sample to the air for 30 min at 4°C (Zhao et al., 2017). A piece of fresh flesh from the Longissimus lumborum was removed after 24 h of refrigeration at 0–4°C, and carcass marbling score was determined by trained individuals through comparison of visual marbling of the Longissimus lumborum using official USDA marbling photographs (National Cattlemen's Beef Association, Centennial, CO, USA) and a 5‐point scale. On this scale, 1 was related to bad and 5 to a very good level of the traits. The pH of meat between the third and fourth lumbar vertebrae was determined at 45 min and 24 h after slaughter. A 5‐cm section of meat was removed from the Longissimus lumborum at the first lumbar vertebra and placed on a clean rubber pad. A circular sampler with a diameter of 2–3 cm (area of approximately 5 cm^2^) was used to cut a 1‐cm‐thick section of the eye muscle and immediately weighed using a weighing balance (sensitivity of 0.001 g). The sample was then placed on and covered with 18 layers of medium‐speed qualitative filter paper with a high‐water absorption capacity. A plastic plate was also placed on the top and bottom, followed by the application of a pressure of 35 kg for 5 min. The pressure was released, and the meat sample was weighed immediately. Drip loss was calculated as a percentage of the amount of water loss of meat samples after being hung in a refrigerator at 4°C for 24 h to the initial weight of the samples (1 cm × 1 cm × 2 cm) (AOAC, 1984). The Warner–Bratzler shear force (WBSF) was measured at the 12th to 13th ribs according to the method of Honikel (1998). The slices were cut perpendicular to the fiber direction with a shearing device (C‐LM3B; Runhu Instrument Co., Ltd., Guangzhou, China). The odor evaluation of meat (20 min in a boiling water bath with 0.5% salt) was performed by a taste panel consisting of 20 panelists using a 5‐point scale (5 = excellent, 1 = very poor). All these traits were evaluated by the panelists and data were recorded based on their preference (like/dislike) for the samples. The cooking rate of mutton was determined by weight loss after cooking the meat for 1 h in a water bath at 80°C, using the formula cooked meat rate = (weight after steaming/weight before steaming) **×** 100.

### 2.3 Fatty acid analysis

Fatty acids in mutton of Tibetan sheep were determined according to the method described by Juárez et al. (2008) with several modifications. Meat samples were ground in liquid nitrogen; 1.0 g of ground samples was heated with 0.7 ml of 10 mol·L^−1^ KOH solution and 5.3 ml of anhydrous methanol (chromatographically pure) in a water bath at 55°C for 90 min. The tubes and contents were then cooled to room temperature, mixed with 0.58 ml of a 12 mol·L^−1^ H_2_SO_4_ solution to methylate the free fatty acids, and placed in a water bath at 55°C for 90 min. The samples were cooled to room temperature, 3 ml of hexane was added, transferred to a centrifuge tube, and centrifuged at 3000 r/min for 5 min. The supernatant was filtered with a 0.22‐μm nylon syringe into a short‐line vial (Labthink Beijing, Beijing, China) and stored at −20°C for fatty acid methyl esters (FAMEs) analysis. The FAMEs were separated on a Shimadzu GC2010 Plus instrument with a flame ionization detector (FID), a split injector, and an AOC‐20i autosampler and quantification. A Restek FAMEWAX capillary column (0.25 mm × 30 m × 0.25 μm) was used. The oven temperature was initially 140°C for 5 min, then increased to 200°C at a rate of 2°C per minute, then to 230°C at a rate of 6°C per minute, and then the temperature was maintained for 20 min. FAME peaks were identified and quantified by comparison with the retention times of a mixture of 37 FAME standards (Supelco 47,885‐U; Sigma‐Aldrich, St. Louis, MO, USA), which were then serially diluted to five concentrations ranging from 10 to 0.625 g L^−1^. Each sample was analyzed for fatty acids in triplicate.

### 2.4 Gene expression analysis

Total RNA was extracted using the TRIzol method, and concentration was determined by measuring the optical density (OD) values using a nucleic acid protein detector. RNA integrity was determined using a gel electrophoresis test. The NCBI database was used for collecting the gene sequences, and Prime5.0 was used to design and synthesize the primers: *MSTN* (NM001009428.3), *Smad*2 (XM027960887.1), *Smad*3 (XM027971520.1), *Smad*4 (NM001267886.1), *TGFβRI* (XM00400422.6), *TGFβRII* (XM 027957940.1), and *TGFβRII* (XM 027957940.1) (Table 1). The fluorescence quantification and reaction conditions used in this experiment, according to the instructions provided with the FastKing RT Kit (Beijing Tiangen Biochemical Technology Co. Ltd), are shown in Tables 2 and 3. The expression levels of MSTN/Smad mRNA in different muscle tissues of Tibetan sheep were determined using real‐time quantitative PCR with reaction conditions shown in Tables 4 and 5. After determining the annealing temperature, the Architex fluorescence quantification instrument was used to construct the amplification and dissolution curves of the corresponding genes; three independent replicates of each sample were tested.

**TABLE 1:** Primers used in RT‐PCR

Gene	Primer	Primer sequences (5′ to 3′)	Length (bp)	Tm (°C)
*MSTN*	*MSTN‐F*	CACCAAGCAAACCCCAAAGGTTCAG	145	57
	*MSTN‐R*	ATGAGCACCCACAGCGATCTACTA*C*		
*Smad*2	*Smad*2*‐F*	TGTTGGGATGGAAGAAGT	471	50.2
	*Smad*2*‐R*	CTGGAATGGAGTGGGTAT		
*Smad*3	*Smad*3*‐F*	CCCAGCCACCGTCTGCAAGAT	134	52.6
	*Smad*3*‐R*	CCAAGAGGGCGGCGAACTCC		
*Smad*4	*Smad*4*‐F*	GTGGCTGGTCGGAAGGGAT	1405	56.2
	*Smad*4*‐R*	AAGGCTGTGGGTCGGCAAT		
*TGF‐βRI*	*TGF‐βRI‐F*	TGGCAGAGCTGTGAAGCCTTG	77	57
	*TGF‐βRI‐R*	AGCCTAGCTGCTCCATTGGCAT		
*TGF‐βRII*	*TGF‐βRII‐F*	CTGGCCAACAGTGGGCAGGTG	99	57
	*TGF‐βRII‐R*	CGTCTGCTTGAAGGACTCGACATT		
*GAPDH*	*GAPDH‐F*	GCGAGATCCTGCCAACATCAAGT	105	60
	*GAPDH‐R*	CCCTTCAGGTGAGCCCCAGC		

**TABLE 2:** PCR reaction mixture composition

Reagent	Dosage (μL)
5 × fastking‐RT Super Mix	4
Total RNA	10
RNase‐free ddH2O	6
Total	20

**TABLE 3:** PCR reaction conditions

Reaction	Temperature	Time
Genomic DNA removal and reverse transcription	42°C	15 min
Inactivated enzyme	95°C	3 min

**TABLE 4:** qPCR reaction mixture composition

Reagent	Dosage (μL)
2 × Su PerReal Color PreMix	10
Forward primers and reverse primers	Each 0.6
cDNA Template	0.5
RNase‐free ddH2O	6.3
Total	20

**TABLE 5:** qPCR reaction conditions

Reaction	Temperature	Time
Pre‐degeneration	95°C	15 min
Degeneration	95°C	30 s
Annealing	52–57°C	30 s
Extension	72°C	5 min
Analysis of melting curve		

### 2.5 Statistical analysis

All quantitative data obtained in the present study were analyzed using one‐way analysis of variance (ANOVA) by SPSS (version 22.0). The mRNA expression of main transduction genes in the MSTN/Smad signaling pathway was estimated by 2^–ΔΔCT^. The multiple comparison procedure was conducted using Duncan's test, and the correlation analysis was analyzed by Pearson's two‐sided test. The results were expressed as mean ± standard deviation.

## 3 RESULTS

### 3.1 Quality analysis of Tibetan sheep meat

The shear force of ram meat was significantly higher than that of ewe meat (*p* < .05), but other indexes were not significantly different (*p >* .05). Muscle pH after 45 min of slaughtering, marbling, odor, and all other indexes were slightly higher in ram meat than in ewe meat, but no significant difference was observed (*p >* .05) (Table 6).

**TABLE 6:** The results of mutton quality analysis of Tibetan sheep

Meat quality index		Ram	Ewe
Meat color	L*	33.10 ± 3.40	37.17 ± 6.53
	a*	14.52 ± 2.77	14.85 ± 3.20
	b*	5.66 ± 1.42	6.66 ± 2.58
pH	45 min	4.08 ± 0.51	3.88 ± 0.68
	24 h	4.67 ± 0.99	5.83 ± 0.23
Drip loss (%)		3.32 ± 0.82	5.56 ± 1.82
Rate of meat cooking (%)		61.07 ± 3.42	62.73 ± 3.14
Shear stress (N)		28.53 ± 8.96^a^	24.99 ± 7.93^b^
Marbling		3.33 ± 0.58	3.33 ± 0.58
Odor		Odorless	Odorless

*Note*: *At .05 level (bilateral); ^a,b^ means in the same column not sharing a common superscript are significantly different (p < .05).

A total of 21 different types of fatty acids were detected in ewe and ram, including nine types of saturated fatty acids, four types of monounsaturated fatty acids, and eight types of polyunsaturated fatty acids. Significant differences in contents of α‐linolenic acid, arachidonic acid, docosadienoic acid, and xylic acid were found between ewe and ram meat (*p* < .05), but other fatty acids showed no significant differences in their contents (*p >* .05). The proportion of oleic acid was the highest (>0.3%) in both ewe and ram meat, followed by palmitic acid (~0.2%). Lauric acid had the lowest content of 0.0006% in ram meat and 0.0004% in ewe meat (Table 7).

**TABLE 7:** The fatty acid content of mutton in Tibetan sheep

Fatty acid (% of total FA)	Ram	Ewe
Lauric acid (C12:0)	0.06 ± 0.03	0.04 ± 0.01
Myristic acid (C14:0)	3.58 ± 1.68	2.13 ± 3.13
Myristic enoic acid (C14:1)	1.40 ± 0.58	2.44 ± 1.38
Pentadecanoic acid (C15:0)	1.52 ± 0.60	1.06 ± 0.30
Palmitic acid (C16:0)	18.84 ± 4.39	19.28 ± 5.48
Palmitoleic acid (C16:1)	1.52 ± 0.65	1.66 ± 0.62
Heptadecanoic acid (C17:0)	0.60 ± 0.08	0.66 ± 0.27
Heptadecanoic acid (C17:1)	0.96 ± 0.32	0.69 ± 0.26
Stearic acid (C18:0)	12.06 ± 1.65	11.26 ± 3.82
Oleic acid (C18:1n9c)	35.60 ± 6.91	32.86 ± 9.85
Linoleic acid (C18:2n6c)	6.96 ± 2.30	3.67 ± 2.07
γ‐Linolenic acid (C18:3n6)	0.30 ± 0.06	0.27 ± 0.16
α‐Linolenic acid (C18:3n3)	0.40 ± 0.12^a^	0.12 ± 0.08^b^
Tridecanoic acid (C23:0)	0.37 ± 0.12	0.25 ± 0.14
Arachidonic acid (C20:4n6)	0.19 ± 0.06^a^	0.01 ± 0.06^b^
Docosadienoic acid (C22:2)	2.65 ± 0.63^a^	1.41 ± 0.76^b^
Xylic acid (C24:0)	0.26 ± 0.07^a^	0.14 ± 0.09^b^
Eicosapentaenoic acid (C20:5n3)	2.47 ± 1.60	5.32 ± 7.08
Neuritic acid (C24:1)	1.71 ± 0.94	1.20 ± 0.41
Docosahexaenoic acid (C22:6n3)	2.23 ± 2.68	4.54 ± 4.82
Heptadecanoic acid (C21:0)	0.35 ± 0.13	0.27 ± 0.15

*Note*: ^a,b,c^Means in the same column not sharing a common superscript are significantly different (*p* < .05).

### 3.2 Expression levels of the MSTN/Smad signaling pathway‐related transduction genes in different muscle tissues of Tibetan sheep

**FIGURE 1:**
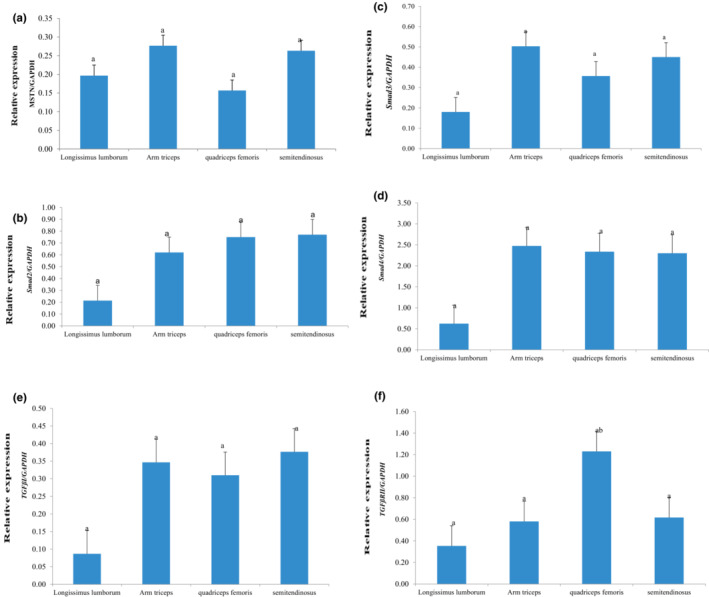
Comparison of relative expression in MSTN/Smad signaling pathway‐associated genes in different muscle tissues of Tibetan sheep. (a) *MSTN*; (b) *Smad*2; (c) *Smad*3; (d) *Smad*4; (e) *TGFβR I*; (f) *TGFβR II*. The lowercase letter shows the results of multiple comparisons in different muscles of the sheep. The same letters represent no significant difference (*p* > .05)

*MSTN*, *Smad*2, *Smad*3, *Smad*4, *Smad*4, *TGFβR I*, and *TGFβR II* genes were found to be ubiquity expressed in four different muscle tissues of Tibetan sheep. The expression levels of *Smad*2, *Smad*3, *Smad*4, and *Smad*4 and *TGFβR I* and *TGFβR II* genes were lower in Longissimus lumborum than other three muscle tissues, but no significant difference was observed (*p >* .05). The expression of the *MSTN* gene showed no significant differences in the arm triceps muscle, quadriceps femoris muscle, semitendinosus muscle, and Longissimus lumborum (*p >* .05) (Figure 1a). The expression of *Smad*2 showed a decreasing order in semitendinosus muscle, quadriceps femoris muscle, arm triceps, and Longissimus lumborum (Figure 1b); the expression of *Smad*3 showed a decreasing order in arm triceps, semitendinosus muscle, quadriceps femoris muscle, and Longissimus lumborum (Figure 1c); the expression of *Smad*4 showed a decreasing order in arm triceps, quadriceps femoris muscle, semitendinosus, and Longissimus lumborum (Figure 1d). The expression of *TGFβR I* was highest in semitendinosus muscle, followed by brachium triceps, quadriceps femoris muscle, and Longissimus lumborum without any significant differences (*p >* .05) (Figure 1e). The expression of *TGFβR II* was highest in quadriceps femoris muscle with a decreasing order in semitendinosus muscle, arm triceps, and Longissimus lumborum (*p >* .05) (Figure 1f).

### 3.3 Correlation analysis of gene expressions in MSTN/Smad signaling pathway and mutton quality in Tibetan sheep

The correlation analysis indicated that gene expressions and meat quality were correlated. In the Longissimus lumborum, we found a significant positive correlation between *MSTN* expression and meat redness (*p* < .05) and a significant negative correlation between *Smad*2 expression and drip loss (*p* < .05, *r* = −1.000). Moreover, the expression of *Smad*3 and *TGFβRII* was significantly negatively correlated with pH_24 h_ (*p* < .05). In the arm triceps, the expression of *Smad*2, *Smad*3, and *TGFβRI* had a significant positive correlation with marbling (*p* < .05). In addition, *Smad*4 expression showed a strong positive correlation with marbling (*p* < .01). In quadriceps femoris muscle, the expression of *MSTN* had a strong negative correlation with the cooking rate of meat (*p* < .01), and the expression of *Smad*2 and *Smad*4 had a significant positive correlation with drip loss (*p* < .05). In *semitendinosus muscle*, the expression of *Smad*3, *Smad*4, and *TGFβRI* had a significant positive correlation with marbling (*p* < .05) (Table 8).

**TABLE 8:** Gene expression correlation analysis between MSTN/Smad signaling pathway genes and meat quality in Tibetan sheep

Mutton quality index	Longissimus lumborum						Arm triceps						Quadriceps femoris muscle						Semitendinosus muscle					
	*MSTN*	*Smad*2	*Smad*3	*Smad*4	*TGF‐βRI*	*TGF‐βRII*	*MSTN*	*Smad*2	*Smad*3	*Smad*4	*TGF‐βRI*	*TGF‐βRII*	*MSTN*	*Smad*2	*Smad*3	*Smad*4	*TGF‐βRI*	*TGF‐βRII*	*MSTN*	*Smad*2	*Smad*3	*Smad*4	*TGF‐βRI*	*TGF‐βRII*
L*	0.434	−0.971	−0.899	−0.985	−0.992	−0.881	−0.289	−0.241	−0.246	−0.218	−0.259	−0.244	0.203	0.945	0.806	0.961	0.990	0.983	0.319	−0.309	−0.174	−0.231	−0.250	−0.139
a*	0.831*	0.282	−0.400	−0.130	−0.087	−0.435	0.345	0.980	0.979	0.984	0.976	0.979	0.751	−0.367	0.557	−0.317	0.097	0.143	0.825	0.963	0.991	0.982	0.978	0.995
b*	0.632	−0.830	−0.995	−0.985	−0.976	−0.990	0.138	0.105	0.100	0.128	0.087	0.103	0.594	0.776	0.960	0.808	0.978	0.987	0.686	0.034	0.173	0.115	0.096	0.208
pH_45 min_	−0.708	0.692	0.068	0.342	0.383	0.029	−0.306	0.962	0.963	0.955	0.967	0.962	−0.724	−0.755	−0.111	−0.719	−0.373	−0.330	−0.801	0.979	0.941	0.959	0.964	0.928
pH_24 h_	0.445	−0.729	−0.998*	−0.943	−0.928	−1.000*	0.596	0.263	0.259	0.286	0.246	0.261	0.907	0.664	0.992	0.703	0.932	0.948	0.951	0.195	0.329	0.274	0.255	0.363
Drip loss (%)	0.442	−1.000*	−0.755	−0.908	−0.925	−0.730	0.094	−0.483	−0.487	−0.462	−0.499	−0.485	0.558	0.997*	0.626	1.000*	0.921	0.902	0.653	−0.544	−0.422	−0.474	−0.491	−0.390
Cooking rate of meat (%)	−0.958**	−0.062	−0.688	−0.463	−0.421	−0.716	−0.878	0.853	0.850	0.865	0.843	0.852	−1.000**	−0.028	0.807	0.025	0.430	0.472	−0.993	0.814	0.887	0.858	0.848	0.903
Shear force (N)	−0.163	0.826	0.271	0.527	0.564	0.234	−0.579	0.885	0.887	0.874	0.893	0.886	−0.898	−0.873	−0.095	−0.846	−0.556	−0.516	−0.944	0.916	0.851	0.882	0.889	0.832
Marbling	−0.162	0.439	−0.240	0.038	0.082	−0.881	−0.579	0.999*	0.994*	1.000**	0.996*	−0.244	−0.898	−0.518	0.410	−0.472	−0.072	0.983	−0.944	0.995	0.997*	1.000*	0.997*	−0.139
Odor	−0.016	−0.016	−0.792	−0.899	−0.659	−0.589	0.523	0.523	0.349	0.246	0.342	0.656	0.052	0.052	0.716	0.806	0.347	0.496	−0.068	−0.068	−0.152	−0.174	−0.173	−0.534

*Note*: **Represents significant correlation (*R*) at .01 level (bilateral); *Represents at .05 level (bilateral).

### 3.4 Correlation analysis of the MSTN/Smad signaling pathway‐associated transduction genes in Tibetan sheep

The correlation analysis of the relative gene expression levels associated with the MSTN/Smad signaling pathway in Tibetan sheep showed that *MSTN* gene expression was positively correlated with *Smad*2, *Smad*3, and *Smad*4 genes and negatively correlated with the *TGFβRII* gene. Other genes also showed a positive correlation but without any significance (*p >* .05). The expression of *TGFβRI* was significantly negatively correlated with *Smad*3 and *Smad*4 gene expressions (*p* > .05) (Table 9).

**TABLE 9:** Relative gene expression correlation analysis of MSTN/Smad signaling pathway genes in Tibetan sheep

	*MSTN*	*Smad*2	*Smad*3	*Smad*4	*TGF‐βRI*	*TGF‐βRII*
*MSTN*	1					
*Smad*2	0.164	1				
*Smad*3	0.66	0.809	1			
*Smad*4	0.336	0.943	0.929	1		
*TGF‐βRI*	0.467	0.946	0.952*	0.970*	1	
*TGF‐βRII*	−0.565	0.68	0.247	0.587	0.445	1

*Note*: **Represents significant correlation (*R*) at .01 level (bilateral); *Represents at .05 level (bilateral).

## 4 DISCUSSION

Tibetan sheep in Qinghai are grazed naturally and provide meat and milk for income to the locals (Xin et al., 2011). Although there is little difference between breeds, Tibetan sheep meat is superior to Small‐tailed Han sheep meat (Jiao et al., 2020). Tibetan sheep meat has good quality with pH_24 h_ between 5 and 6, meat cooking rate between 25% and 28%, and drip loss between 4% and 6% (Zhang et al., 2022), which are similar to the results of this study. We also observed that the shear force of ram meat was significantly higher than that of ewe meat (*p* < .05). Furthermore, Tibetan sheep meat contains a good proportion of diverse kinds of fatty acids (Huang, 2015). Further, the fatty acid composition of adipose tissue and muscle determines the oxidation stability of adipose tissue and muscle hardness, thus affecting the flavor, tenderness, and color (Wood et al., 2008), and it is one of the most important factors affecting meat quality. Bao et al. (2021) have identified 26 different fatty acids in the Longissimus thoracis muscle of Tibetan sheep, including ten different types of saturated fatty acids (SFA), seven different types of monounsaturated fatty acids (MUFA), and nine different types of polyunsaturated fatty acids (PUFA). Wang et al. (2021) have assessed the meat quality of Urumqi sheep, Hu sheep, and Tibetan sheep, and found that Tibetan sheep meat contains the highest amounts of polyunsaturated fatty acids and unsaturated fatty acids, which is consistent with our results. A total of nine different types of saturated fatty acids, four different types of monounsaturated fatty acids, and eight different types of polyunsaturated fatty acids were detected by us in Tibetan sheep muscle. In addition, we observed a significant difference in the content of linolenic acid, arachidonic acid, docosadienoic acid, and xylic acid between ram and ewe meat.

*MSTN* is a member of the TGF‐β family, and the *MSTN* signaling pathway consists of *TGFβRI*, *TGFβR II*, and Smad proteins similar to the TGF‐β/Smad signaling pathway that regulates skeletal muscle growth (Kollias & McDermott, 2008; Wang et al., 2016; Yao et al., 2016). Mateescu & Thonney (2005) have shown that *MSTN* is expressed in the semitendinosus muscle of Dorset‐sired ram lamb. The relative expression of *MSTN* in leg muscle is higher than that in the stomach, rumen, and cardiac muscle tissues of the Tibetan sheep (Liang et al., 2011). Xu has discovered that *Smad*2 and *Smad*4 are expressed in the heart, liver, spleen, lung, kidney, hypothalamus, pituitary, and muscle tissues of Hu sheep (Xu, 2010). Gao et al. (2016) have uncovered that the gene expressions of *Smad*2, *Smad*3, and *Smad*4 in Longissimus lumborum and soleus muscle tissues of 2‐day‐old Hu sheep are significantly higher than those in other age groups. Anwaier has revealed that *Smad*2, *Smad*3, and *Smad*4 of Turpan black sheep are expressed in biceps femoris, triceps femoris, longissimus dorsi, semitendinosus, and leg muscles (Anwar et al., 2016). However, *TGF‐βRI* and *TGF‐βR II* are not detected in Hu sheep muscle tissues (Xu, 2010). *TGF‐βRI* and *TGF‐ΒRII* show low levels of expression in the muscle tissue of Turpan black lamb (Anwar et al., 2016). In the present study, the main MSTN/Smad signaling pathway genes of Tibetan sheep were found to be expressed in Longissimus lumborum, arm triceps, quadriceps femoris muscle, and semitendinosus muscle without any significant difference.

The correlations between the gene expressions in the MSTN/Smad signaling pathway and meat quality of Tibetan sheep were analyzed in this study, and a significant correlation was found between gene expression in muscle tissues and meat redness, pH_24 h_, drip loss, meat cooking rate, and marbling. Meat color is one of the most important physical indicators of meat quality and is directly related to the content of myoglobin (Kim et al., 2010). *MSTN* expression in the Longissimus lumborum of Tibetan sheep was found to be significantly correlated with meat redness. Muscle pH is an important index for determining the rate of glycolysis in muscles after slaughter (Zang, 2010). Previous research has also discovered that the decreasing pH value affects meat quality (Bertol et al., 2013). In our research, we found that *Smad*2 and *TGFβRII* genes in Longissimus lumborum had a significant correlation with pH_24 h_ in Tibetan sheep. The water‐holding capacity and cooking rate of meat affected both yield and product quality and were correlated with the tenderness, juiciness, and nutritional value of the meat (McKenna et al., 2005). The expression of *MSTN* and *Smad*2 in longissimus dorsi and quadriceps femoris muscles was found to be significantly correlated with drip loss and the cooking rate of meat. Marbling and tenderness determine the juiciness and freshness of meat, which are important indicators of meat quality and taste (Liu, 2007). This study concluded that *Smad*2, *Smad*3, *Smad*4, and *TGFβRI* genes in arm triceps and semitendinosus muscle were significantly correlated with marbling. The MSTN/Smad signaling pathway plays an important role in the regulation of postnatal skeletal muscle growth, development, and repair in mammals, such as sheep and cattle (Kollias & McDermott, 2008), and *MSTN* inhibits skeletal muscle growth and development (Elliott et al., 2012). When TGF‐β binds to *TGFβRII*, the *TGFβRI* receptor is activated, which stimulates the phosphorylation of *Smad*2 and *Smad*3. Further, activated *Smad*2 and *Smad*3 form a transcriptional complex with *Smad*4, regulating the transcription of target genes by interacting with transcription factors, repressors, or activators in the nucleus. Smad7 protein binds tightly to *TGFβRI* and inhibits TGF‐β and the phosphorylation of *Smad*2 and *Smad*3; therefore, TGF‐β has a negative feedback impact on the regulation of target gene transcription (Li et al., 2008). Furthermore, Droguett et al. have found increased expression of *TGFβRI* and *TGFβRII* during myogenesis, and downregulation of *Smad*2, *Smad*3, and *Smad*4 expression during skeletal muscle differentiation. The translocation of Smad3 and Smad4 proteins to the nucleus is inhibited by TGF‐β after skeletal muscle differentiation, which allows TGF‐β to affect gene expressions of *Smad*2, *Smad*3, and S*mad*4 at the intracellular level in myoblasts. Therefore, TGF‐β plays an important role in the feedback regulation of target gene transcription (Droguett Mallea et al., 2010; Serizawa et al., 2013).

In the current study, we concluded that *MSTN* expression was positively correlated with *Smad*2, *Smad*3, *Smad*4, and *TGF‐βRI* gene expressions but negatively correlated with the expression of *TGF‐βRII*. At different muscle ages (0–6 months), *MSTN* expression has a positive correlation with *Smad2* expression in Altay sheep and Turpan black sheep muscles (*p* > .05), and *Smad*3 expression is positively correlated with *Smad*4 expression (Anwar et al., 2016). Similar results were observed in our study. However, *MSTN* expression is negatively correlated with *Smad*2 expression in the Longissimus lumborum of Hu sheep (Gao et al., 2016), which was inconsistent with the findings of this study. Hence, specific reasons and mechanisms leading to these correlation differences require further experimentation.

## 5 CONCLUSIONS

In conclusion, this study demonstrated an association between meat quality and the expression of the major MSTN/Smad signaling pathway‐associated genes in the muscle of Tibetan sheep. This study also revealed that the expression of *MSTN* was significantly correlated with the redness and cooking rate of meat. Moreover, *Smad*2 and *Smad*4 expression showed a significant correlation with drip loss and marbling; *Smad*3 expression had a significant correlation with pH_24 h_ and marbling; *TGFβRI* expression had a significant correlation with marbling, and *TGFβRII* expression had a significant correlation with pH_24 h_. The correlation of main transduction gene expression was analyzed and revealed a negative correlation between *MSTN and TGFβRII* gene expressions, while other genes exhibited a positive correlation. Moreover, *TGFβRI* expression was significantly correlated with the expression of *Smad*3 and *Smad*4. The results of this study provide a theoretical foundation to improve the meat quality and breeding process of Tibetan sheep.

## FUNDING INFORMATION

This work was supported by the funds of the Science and Technology Planning Program of Qinghai (Science and Technology Department of Qinghai Province) (Grant No. 2020‐ZJ‐786); Outstanding person of Kunlong: rural revitalization Program (Grant No. (2020)9); The program of the Gansu Provincial Key Laboratory of Cattle and Sheep Germplasm and Straw Fodder, Gansu Academy of Agricultural Sciences (2019KL01); The project of Research Condition Construction and Achievement Transformation of Gansu Academy of Agricultural Sciences (Modern Biological Breeding, 2021GAAS01).

## CONFLICT OF INTEREST

We certify that no financial organization has a conflict of interest concerning the material discussed in this manuscript.

## ETHICS APPROVAL

All animal procedures were carried out under the guidelines of the Regulation of the Standing Committee of Qinghai People's Congress. All experimental protocols and the collection of samples were approved by the Ethics Committee of Qinghai University under permission no. SL‐2021027.

## Data Availability

The data that support the findings of this study are available from the corresponding author upon reasonable request.
